# Innovative colorimetric thermal study of methylcellulose hydrogel via smartphone imaging

**DOI:** 10.1038/s41598-025-00018-1

**Published:** 2025-05-04

**Authors:** Itai Danieli, Daniel Rittel

**Affiliations:** Faculty of Mechanical Engineering, Technion – Israel Institute of Technology, Haifa, 3200008 Israel

**Keywords:** Novel methodology, Methylcellulose, Gels, Colorimetry, Gelation., Engineering, Mechanical engineering, Materials science, Materials for optics, Soft materials, Techniques and instrumentation, Optics and photonics, Optical techniques

## Abstract

A novel colorimetric analysis of methylcellulose (MC) hydrogel was conducted using a standard smartphone camera to measure its thermo-optical properties. As demonstrated for the first time, the temperature of MC gels was directly determined from photographs by exploiting a unique one-to-one correlation between temperature and mean pixel intensity in the blue channel of RGB images, all color channels showed strong hysteresis. Two innovative procedures for gelation assessment are introduced: variance analysis and histogram analysis with normal distribution fitting. The variance analysis confirms known gelation related temperatures, validating the effectiveness of the new method. Histogram analysis reveals a significant increase in RMSE (root mean square error) near gelation related points, offering a new indicator for gelation status. This methodology underscores the untapped potential of colorimetry to extract valuable data from hydrogels in general and MC gel in particular.

## Introduction

Colorimetry quantifies human color perception^1^. According to the trichromatic theory, humans distinguish colors using three types of receptors sensitive to wavelengths in varying manner^2^. Digital imaging (RGB) mimics this perception, ensuring that phenomena visible to the human eye can be captured by RGB sensors^3^. In that respect, whereas the human eye provides high quality qualitative information, a sensor provides a wealth of data points that can be analyzed and sorted, as discussed in the sequel. Colorimeters measure the amount of light transmitted through or reflected by materials, via analysis simulating the human vision^4^. These devices are commonly used in chemical and bioanalytical analysis^5–7^, such as measuring metal concentrations^8–10^, and monitoring blood glucose levels^11,12^. For the specific case of MC hydrogel, previous studies have shown that this gel gradually changes its optical properties with temperature, visible to the naked eye^13–15^. This suggests that the correlation between MC gel’s optical properties in the visible spectrum and its temperature can be analyzed using colorimetry. The motivation of this research stems from the lack of non-contact temperature measurement methods for water-based, thermochromic gels, which are gels that exhibit distinct color response with temperature changes, such as MC. Since water absorbs IR radiation, traditional IR sensing methods may fail to provide useful results. Therefore, in addition to the inherent findings of the study which enhance the understanding of the thermo-optical properties of MC gels. We demonstrate the efficacy of the colorimetry method using a camera and highlight its significant potential for further analyzing the color-coupled behavior of MC gels. MC Which are being considered for bodily protection^14,16,17^, and smart biomaterials^18–20 ^among their many applications and advancements in the food and medicine industry^13,21–24^.

Calorimetric evaluations were conducted to quantify the energy flux of methylcellulose (MC) gels during both heating and cooling cycles, thereby elucidating their gelation behavior. Specifically, gelation upon heating is characterized by a distinct endothermic process with peak heat flow at approximately $\:62\left[{C}^{o}\right]$, while a broader and less pronounced exothermic process with peak at around $\:32\left[{C}^{o}\right]$during cooling^25–30^. The onset of the gelation process occurs at lower temperatures and progresses to higher temperatures as the concentration increases^26,28^. In contrast, the extremums appears to remain largely invariant across different molecular weights and concentrations, as detailed in^26,27^and substantiated by graphical interpretations in^25,28,30^. Optical analyses of MC gels have revealed abrupt changes in turbidity at selected wavelengths, manifesting as step-like transitions in turbidity measurements at 500 nm, 545 nm, and 660 nm^26,27,31^. These transitions are closely aligned with the corresponding endothermic and exothermic peaks noted in the calorimetric studies^26,27^. Furthermore, an investigations across various types and concentrations of MC upon heating demonstrated that while concentrations up to $\:2\:wt\%\:$affect optical transmittance behavior of 545 nm wavelength light, this effect plateaus at higher concentrations. In those, turbidity begins to increase at approximately $\:45\left[{C}^{o}\right]$, with the cloud point temperature consistently observed near $\:62\left[{C}^{o}\right]$^31^. The heat flow extremum associated with gelation, which corresponds to the changes in turbidity at the wavelengths mentioned before, appears to be minimally influenced by temperature ramp variations ranging from $\:\frac{1}{6}\left[\frac{{C}^{o}}{min}\right]$ to $\:1\left[\frac{{C}^{o}}{min}\right]$for both heating and cooling cycles^26^. A substantially faster heating ramp of $\:10\left[\frac{{C}^{o}}{min}\right]$ results in an approximate $\:5\left[{C}^{o}\right]$shift in the observed extremum relative to the slower ramp conditions previously discussed^30^.

In addition to gelation, MC is known to undergo phase separation. At concentrations exceeding $\:0.5\:wt\%$, phase separation is observed at temperatures above those corresponding to gelation^26,28^. Although it has been suggested that MC does not undergo phase separation below $\:80\left[{C}^{o}\right]$^26^, there are reports indicating occurrence of the phenomena at temperatures slightly above $\:60\left[{C}^{o}\right]$for some MC types^27^.

Assessments of light transmission at 460 nm in MC have revealed increases and decreases during both the heating and cooling processes, a behavior that contrasts with the step-like transmittance changes observed at the previously mentioned wavelengths^32^. This difference, and the gradual color change of MC gels with temperature variation as interpreted by the naked eye suggest that the optical properties of MC gels are reflected over a broader range of wavelengths, as opposed to a single wavelength. The qualitative turbidity changes of MC gel upon heating is well documented, Parvari et al.^14^showed a similar transition under impact loading. Those authors also carried out a preliminary semi-quantitative analysis of the degree of turbidity resulting from gel heating to compare with their impact results. Other contactless methods have also been successfully used for analyzing MC, Raman spectroscopy^33^, UV–vis spectroscopy and test tube tilting method^34^. Static light scattering techniques were also used for assessing the thermoreversible gelation of gelatin^35^. However, a comprehensive study about analyzing MC thermodynamics using a standard camera for colorimetry has yet to be performed, as presented in this work. This is a simple, cost-effective, and contactless method for analyzing the temperature and sol-gel transitions of MC gels. This method may also be applicable to various gels that undergo visible changes with temperature and that undergo phase transitions.

## Materials and methods

### Preparation of MC hydrogel

MC gel was formulated by incorporating MC powder (SG A7 C food grade, DOW chemicals) into hot, purified water at a minimum temperature of $\:65\left[{C}^{o}\right]$. The water weight was measured after heating to minimize evaporation during heating. The mixture was stirred until a homogenous solution was obtained. Subsequently, the beaker holding the hot MC mixture was immersed in cold water to activate the gel formation. The prepared $\:5.2\left[\%\right]$ weight MC hydrogel was stored in a refrigerator $\:\left(1-4\left[{C}^{o}\right]\right)$overnight (at least 12 h) and kept in refrigerator until experiments were done^17^.

### Experimental setup

**Fig. 1:**
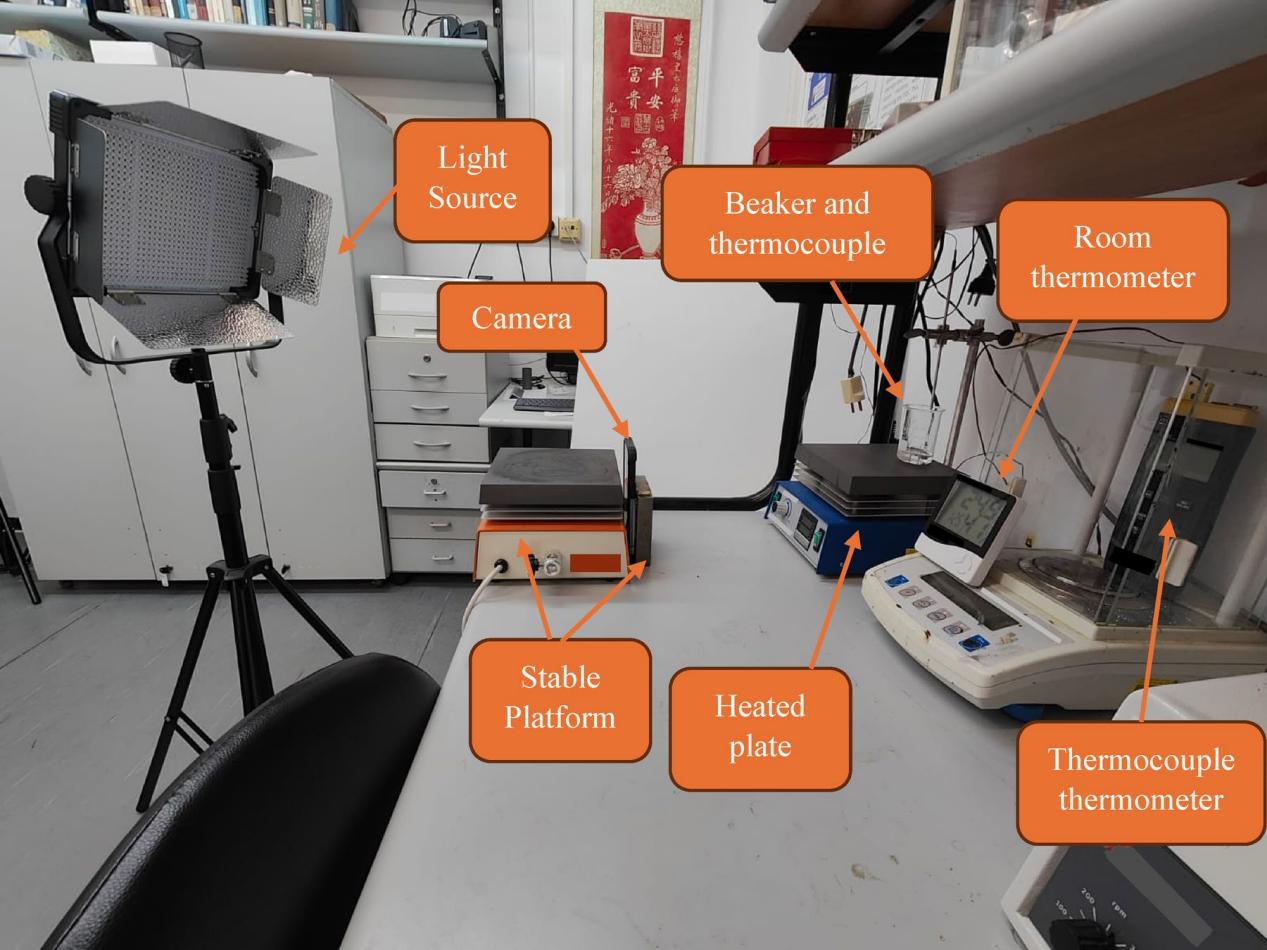
Experimental setup.

The imaging setup is shown in Fig. 1.

In Fig. 1, a beaker (filled with MC during experiment) was placed on a heated plate. A thermometer and thermocouple were used for temperature measurement. The photographic data was captured using an iPhone 12 Pro Max camera utilizing the “ProCamera” app^36^, which allows for comprehensive manual control over the camera settings and for photo acquisition using timer, those ensure consistent precision. The camera was set to capture images in TIFF mode to preserve the maximum amount of image information. White balance, focus and exposure settings remained constant throughout the experiment. The thermocouple thermometer used is a Lutron Thermometer (3 in 1) - TM-2000, type K thermocouple was employed to continuously monitor the temperature. A relatively strong LED light source (Yongnuo YN9000) was used to minimize inconsistencies from disturbing light sources such as ceiling lights and sunlight. Ambient temperature measured by the room thermometer was between: $\:24\left[{C}^{o}\right]-26\left[{C}^{o}\right]$.

**Fig. 2:**
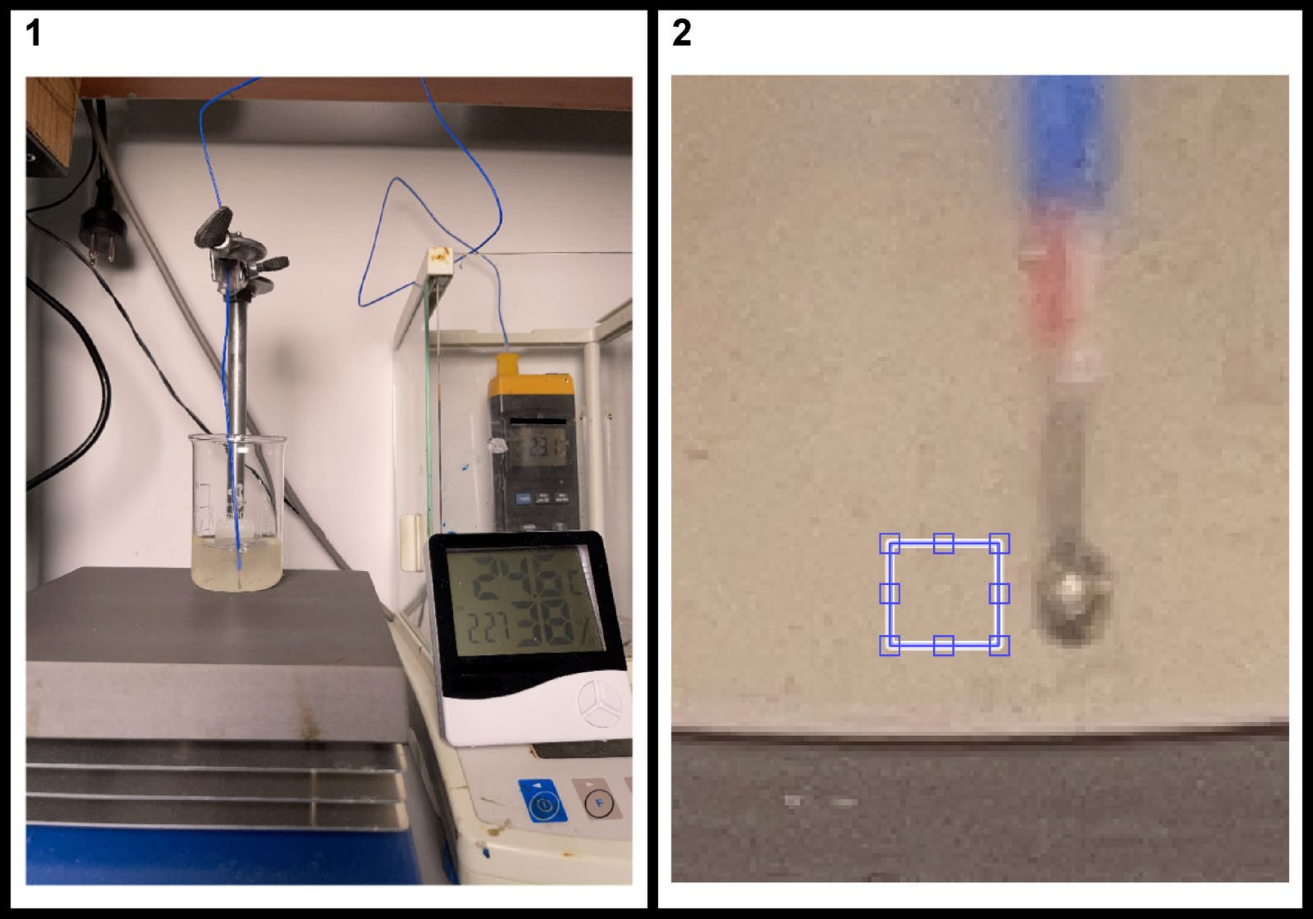
Example of photo analyzed. Whole photo [1] and zoomed photo [2]. Blue rectangle in the zoomed photo is the area being analyzed.

The whole photos were not analyzed, as a small $\:<O\left(10\left[{mm}^{2}\right]\right)$ section of the MC close to the thermocouple probe was used, as it gave meaningful results. This could be for the small section minimized inconsistencies of lightning and thickness of MC (cylindrical beaker). In Fig. 2 one can see an example photo and the analyzed part.

Since the experimental system remained still (besides cooling and heating), the area being analyzed is the same one for all the photos. The area being analyzed is $\:24 x 22$ pixels. Where the length of the side of each pixel is approximated to be:

$$\:{d}_{pixel}\approx\:0.105\left[mm\right]$$

### Statistical analysis

The statistical analysis was conducted using MATLAB^37^, utilizing its built-in functions for various calculations. Simple statistics such as variance and mean were computed using the appropriate MATLAB functions. Normal distribution fitting was performed using the “fitdist” function^38^. The smooth dataset was produced using the “smooth” function^39^. The code used for analysis is given in the research data. Statistical analysis was performed on each photo separately, excluding “smooth” that was used on the values between photos in the same temperature, as “smooth” function result is the mean value for each temperature group.

## Results

### Mean pixel intensity as a function of temperature

**Fig. 3:**
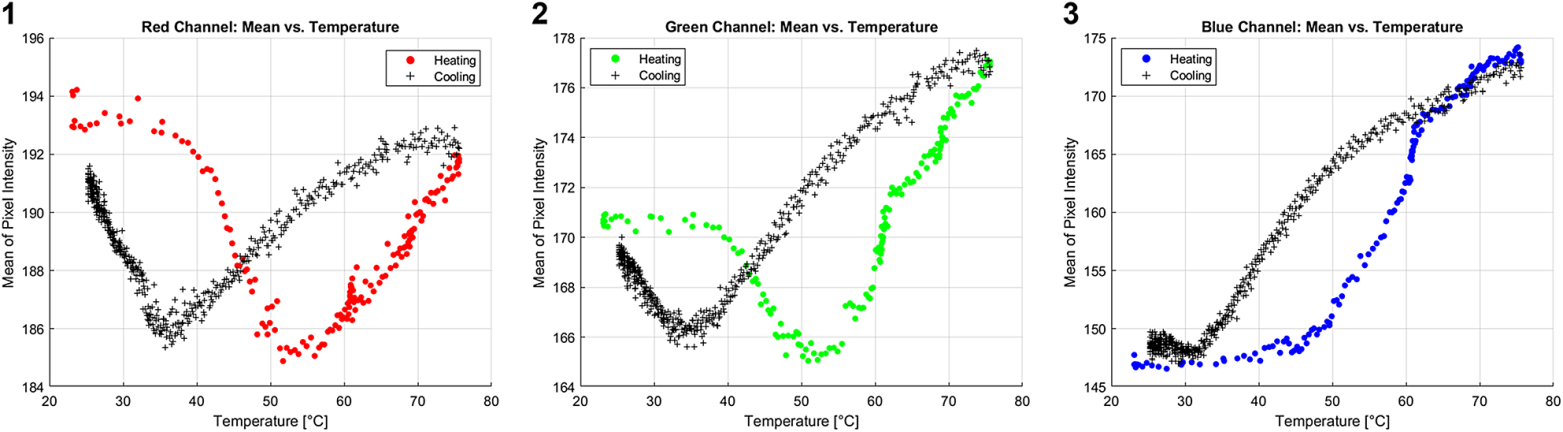
Temperature vs. mean pixel intensity for red [1], green [2],and blue [3] channel - heating and cooling.

The MC underwent a thermal cycle, consisting of a heating phase followed by a cooling phase. The heating process was completed in approximately 20 min, while the cooling phase extended beyond one hour. Images were captured every seven seconds, with a slight delay for image processing. Mean pixel intensity of analyzed photo was plotted against temperature, as shown in Fig. 3.

Figure 3shows a clear sensitivity of the camera to temperature changes of MC gel. The blue channel showed a classical hysteresis response in most temperature ranges, while the red and green channels who do not have one on one response yet exhibited a strong hysteresis response.

### Variance of pixel intensity as a function of temperature

**Fig. 4:**
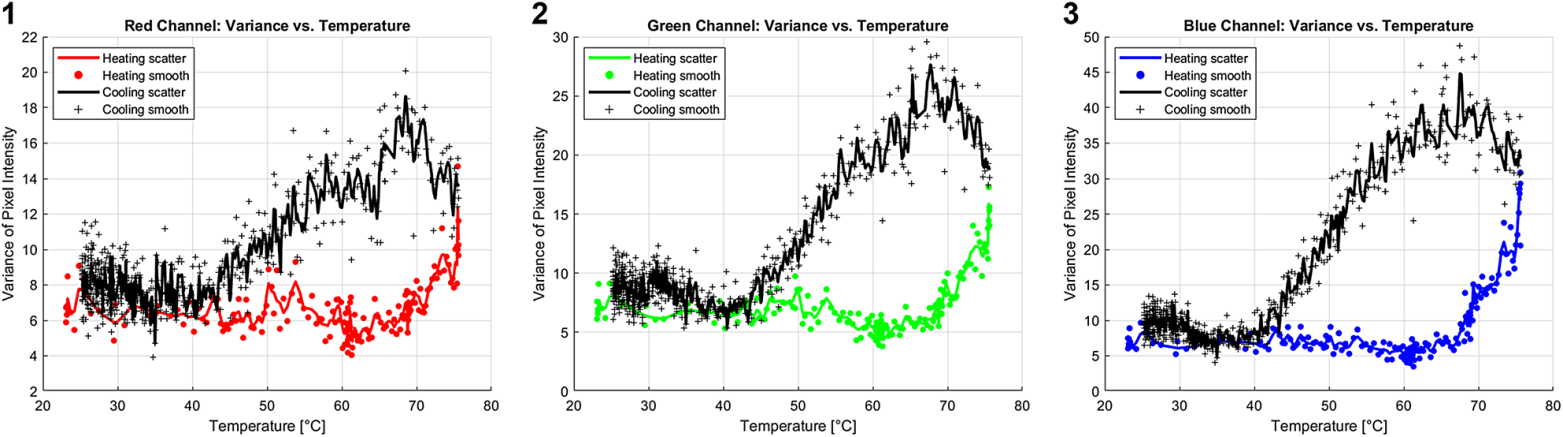
Temperature vs. Variance of Pixel Intensity For red [1], green [2], and blue [3] channels. Scatter dots are the results from each experiment, smooth line is the mean result of experiments at each temperature.

Variance of pixel intensity of the analyzed area was plotted against temperature, as shown in Fig. 4:

Figure 4 shows that the variance of pixel intensity is relatively steady upon heating, and that around $\:65\left[{C}^{o}\right]$ it starts to increase significantly. It continues to rise a little upon cooling and then returns to its original value around $\:35\left[{C}^{o}\right]$ for the blue channel and $\:40\left[{C}^{o}\right]$ for the green and red channels. Those results are close to those attributed to extremums of calorimetry analysis of MC and rapid change in turbidity. Thus, showing that via variance analysis of camera-based colorimetry, one can gain insights on the gelation process of MC gels, where a sharp increase in variance upon heating indicates that the MC is undergoing its most intense endothermic process. Conversely, as the variance returns to its initial value during cooling, it signifies that the MC is experiencing its most intense exothermic process.

### Histogram analysis

**Fig. 5:**
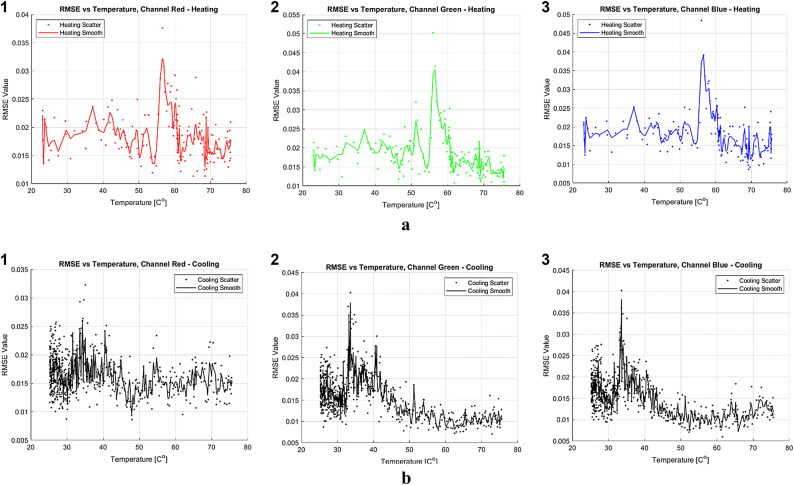
**a** Analysis of RMSE values for heating of normal distribution fit across RGB channels as a function of temperature. Each subplot represents a distinct color channel and order, illustrating the fit of a normal distribution to the pixel value distributions. Subplots 1–3 show the process for the red, green, and blue channels, respectively. Scatter dots represent the results from individual experiments, while the smooth line indicates the mean result of experiments at each temperature.** b **Analysis of RMSE values for cooling of normal distribution fit across RGB channels as a function of temperature. Each subplot represents a distinct color channel and order,illustrating the fit of a normal distribution to the pixel value distributions. Subplots 1–3 show the process for the red, green, and blue channels, respectively. Scatter dots represent the results from individual experiments, while the smooth line indicates the mean result of experiments at each temperature.

**Fig. 6:**
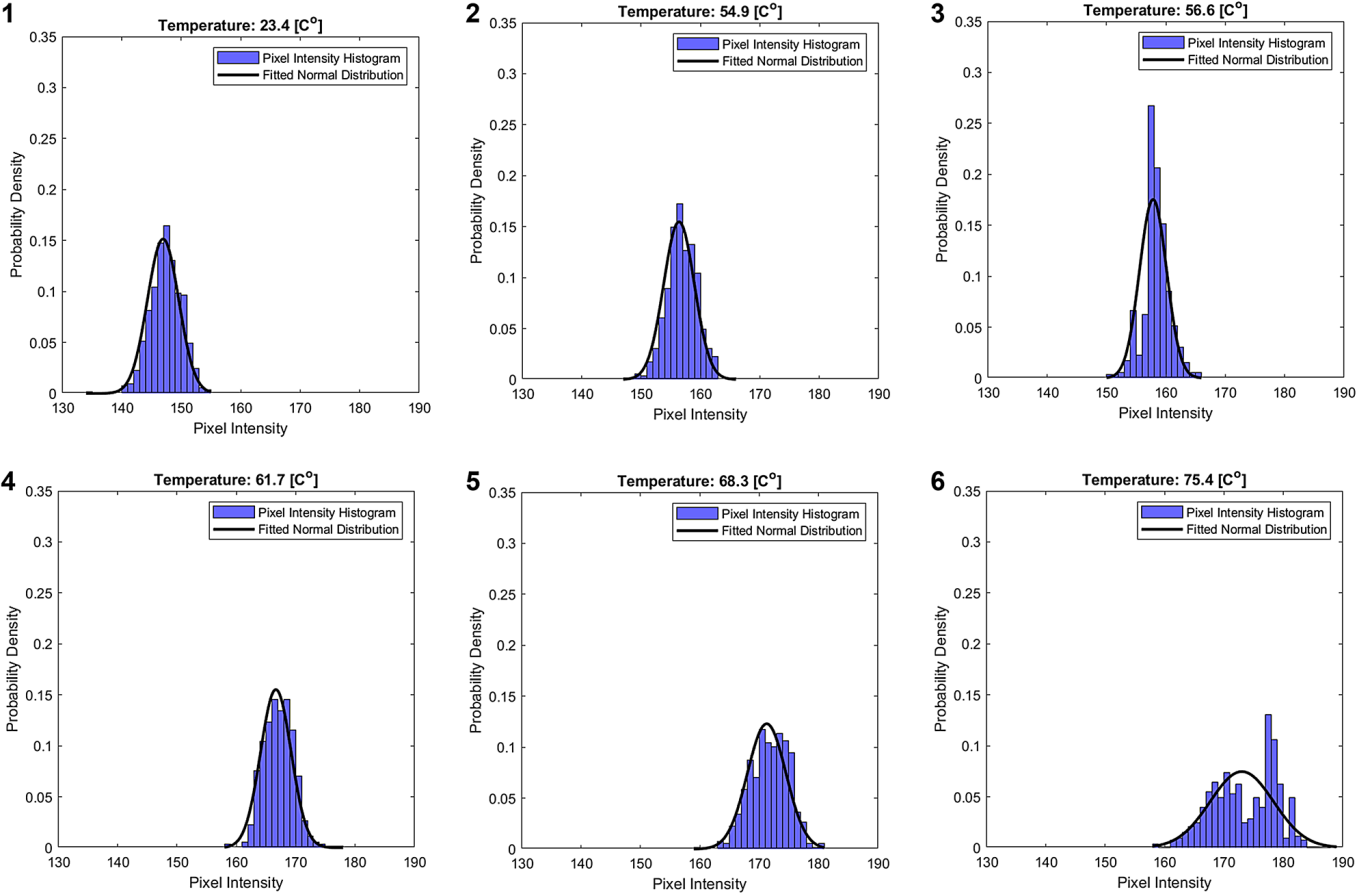
Histograms of pixel intensity for the blue channel upon heating at selected temperatures. Subplots 1,*2* $\:(23.4\left[{C}^{o}\right],\:54.9\left[{C}^{o}\right]$, *Subplot 3* $\:(56.6\left[{C}^{o}\right]$, *Subplot 4* $\:(61.7\left[{C}^{o}\right]$, *Subplot 5* $\:\left(68.3\left[{C}^{o}\right]\right)$, *and Subplot 6* $\:\left(75.4\left[{C}^{o}\right]\right)$.

Data’s histogram was fitted to normal distribution and the RMSE between the histogram bin centers to it (goodness of fit) was plotted against temperature, results can be seen in Fig. 5a for heating and Fig. 5b for cooling:

Figure 5a shows that during heating, RMSE remains relatively constant before peaking at around $\:56\left[{C}^{o}\right]$ upon heating, and according to Fig. 5b there is a broader peak at around $\:33\left[{C}^{o}\right]$ upon cooling. The observed temperatures are similar to those attributed to extremums of calorimetry analysis of MC. Additionally, the broader cooling RMSE peak aligns with previous calorimetric analyses, suggesting that the degelation process occurs over a wider temperature range. This analysis suggests that RMSE values also provide useful insights into the gelation state. The blue channel exhibited the clearest response, likely due to its one-to-one correlation with mean pixel intensity in almost all temperature ranges, as observed in Fig. 3. To gain further insights, probability histograms of the blue channel upon heating, at temperatures of interest are presented, with a fitted normal distribution, as in Fig. 6.

In Fig. 6, subplots 1,2 that correspond to temperatures of $\:23.4\left[{C}^{o}\right]$ and $\:54.9\left[{C}^{o}\right]$ respectively, exhibit a normal. In Subplot 3, at $\:56.6\left[{C}^{o}\right]$, there is a notable sharp increase in RMSE, marked by a higher peak and an asymmetry in the distribution. As the temperature rises to $\:61.7\left[{C}^{o}\right]$ in Subplot 4, the RMSE shows a recovery, with the distribution returning to a more normal shape. Those observations support the findings from Fig. 5, which shows a rapid increase in RMSE near the gelation temperatures. Subplots 5 and 6, corresponding to temperatures of $\:68.3\left[{C}^{o}\right]$ and, $\:75.4\left[{C}^{o}\right]$ respectively, display further structural changes in the distribution. In Subplot 5, the distribution spreads, and in Subplot 6, the presence of two distinct peaks becomes apparent. This phenomenon reinforces the results from Fig. 4, which demonstrated higher variance at temperatures higher than those attributed to gelation, providing additional insights into the factors driving these shifts. Furthermore, the general trend of the histograms shifting to the right as temperature increases corroborates the findings from Fig. 3.

## Discussion

The findings from the experiment conducted provide significant insights into the optical properties of MC in response to temperature variations, as captured through optical imaging. In Fig. 3, the analysis of mean pixel intensity reveals distinct behavior across the color channels. While all channels exhibit a strong hysteresis response, the blue channel displays the closest response to a one-to-one function with temperature changes. The observed response of the color channels is attributed to the inherent properties of MC and to factors related to the experimental setup, such as lighting conditions. As a result, and according to preliminary experiments that were not reported it should not be assumed that the color channels in slightly different experimental setups will behave identically to those observed in this experiment. However, one can expect them to demonstrate sensitivity under appropriate lighting and camera conditions. It is also not recommended to prioritize any specific color channel in advance, nor to apply color space transformations that may lead to the loss of valuable information, such as converting from RGB to grayscale.

**Fig. 7:**
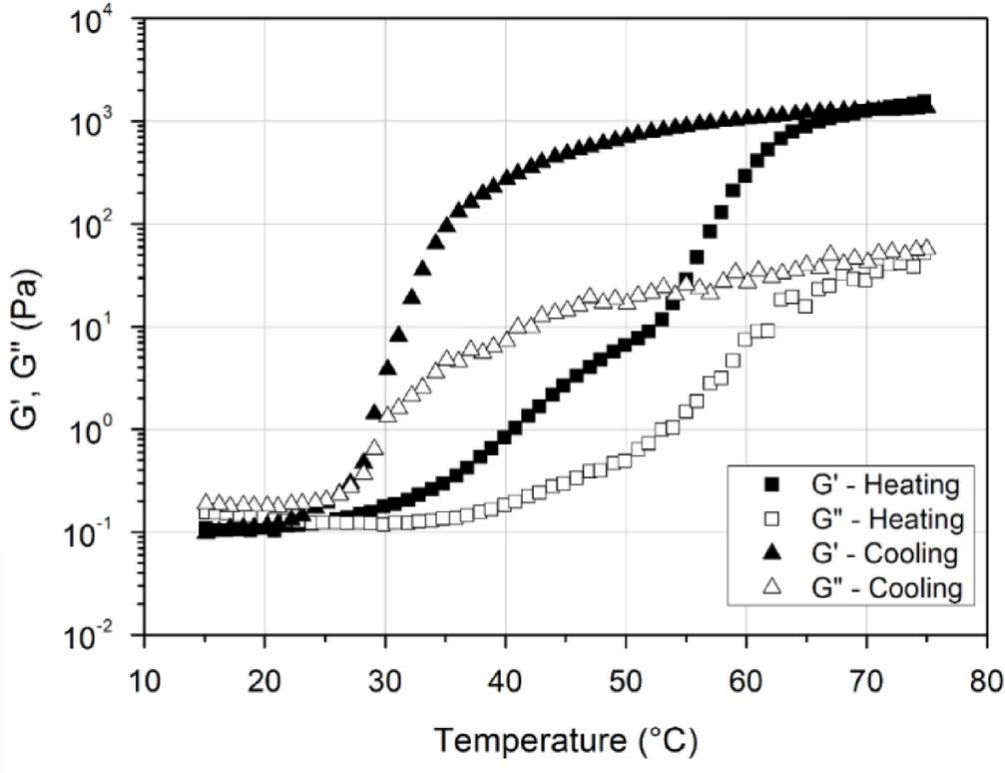
Temperature dependence of dynamic viscoelasticity for methylcellulose (reproduced with permission^15^, 2015, Taylor & Francis).

It was reported that the MC gels show distinctive dynamic viscoelastic behavior that varies with temperature. While this thermomechanical distinct behavior of MC varies a bit for varying concentrations and molecular weights of MC, it remains relatively the same in all the reported research, in which different concentrations and molecular weight of MC were used^15,25,29,40^. Figure 7, reproduced from^15^, shows the storage $\:\left({G}^{{\prime\:}}\right)$ and loss moduli $\:\left({G}^{{\prime\:\prime\:}}\right)$ of MC gel as a function of the temperature. These measurements were taken using a rheometer at a frequency of $\:0.5\left[Hz\right]$, with a temperature ramp of $\:0.5\left[\frac{{C}^{o}}{min}\right]$.

Both bulk and loss moduli display a distinct hysteresis behavior upon heating and cooling. A comparison between the blue channel in Fig. 3.3 and the dynamic viscoelasticity data of MC that presented in Fig. 7 reveals a remarkable correlation. Both the optical and mechanical properties exhibit similar trends in response to temperature changes, highlighting the interconnected nature of MC’s thermo-optical, and thermo-mechanical properties.

Regarding the detection of gelation status, as illustrated in Figs. 4 and 5, increased variance was observed at temperatures slightly higher than those typically associated with extremums of calorimetry analysis of MC and rapid change in turbidity of optical analysis. Additionally, an increase in RMSE was noted around those temperatures during both the heating and cooling processes, with the RMSE peak during cooling being broader, this aligns with previous calorimetric analyses. An increase in RMSE and variance changes serve as an indicator of gelation status. It is important to note, however, that while these findings are compelling, the underlying relationship between pixel intensity variance, RMSE, and the gelation of MC remains unclear. Further research is required to clarify the mechanisms causing changes in RMSE and variance. Similarly to mean analysis advice, the camera should detect changes in the pixel intensity distribution- presented in this paper as variance and RMSE, however, the specific changes might vary. Finally, Fig. 6 provides a qualitative summary of the discussed data, with a focus on the blue channel, which was the most sensitive and demonstrated a one-to-one response to temperature. Notably, this channel also displayed signs of a bimodal distribution, unlike the other channels, likely due to its higher sensitivity and direct temperature response. This structural change may hold significant implications for future research from a theoretical perspective.

For future investigations, it is recommended that the camera be positioned closer to the MC sample to reduce fluctuations in the results. It is also suggested to further verify the method for measuring sol-gel transition on other gels, as it will further validate it and may lead to a better understanding of the mechanism causing the sol-gel transition being detectable to the camera. Additionally, phase separation was not directly monitored. Although the heated bed may have created temperature gradients exceeding $\:60\left[{C}^{o}\right]$- a threshold noted in the introduction for phase separation^27^, the hysteresis behavior suggests that phase separation did not affect the results. Nonetheless, future studies should explicitly monitor for phase separation. Using a camera that has known sensitivity to light is advised, as it will allow to quantify the results with physical coherence and will allow for better advancement in theoretical model of the methodology.

When compared to other contactless methods, camera-based colorimetry offers two significant advantages. First, its cost-effectiveness is notable. Cameras are relatively inexpensive, and nearly every modern smartphone is equipped with a camera that meets the requirements for this analysis, as confirmed by our experimental results. Second, the extensive data captured by cameras facilitates a deeper understanding of the thermal properties of MC. In our study, even a basic analysis successfully identified temperatures correspond to extremums of calorimetry analysis of MC and rapid change in turbidity and the temperature of MC. With more advanced analytical techniques, potentially incorporating machine learning, more detailed and accurate insights could be achieved. Nonetheless, the novelty of this approach introduces certain challenges. Specifically, the results obtained in our laboratory may vary when different cameras or lighting conditions are used. Although this work demonstrates a promising potential of camera-based colorimetry for MC analysis, further, standardization is essential for refining both the temperature measurements and the gelation assessments.

One important point is that the focus of the present study is on a novel non-contact methodology, illustrated on a specific well-documented hydrogel, and not the contrary. Although the study is based on reasonable assumptions supported by the literature, we recommend that future studies adhere to established protocols, particularly regarding MC type, hydrogel concentration, and thermal ramp. Furthermore, pairing colorimetry with validated measuring instruments could significantly enhance its reliability. To summarize, we have shown different, yet complementary, types of analysis, ranging from mean and variance of pixel intensity to histogram analyses. Those methods can measure the temperature of MC and indicate gelation status upon heating and cooling and can be used indistinctly, noting the great advantage of this non-contact method.

## Conclusions

This study highlights the significant influence of temperature on the optical properties of MC as observed through an optical camera. The findings demonstrate that an optical camera can serve as an excellent, novel and affordable tool for monitoring the temperature of MC. Furthermore, by applying statistical analysis to the extensive data provided by the optical camera, deeper insights into the thermodynamics of MC can be obtained and temperatures correspond to extremums of calorimetry analysis of MC and rapid change in turbidity of optical analysis can be measured. The methodology may also apply to various gels that undergo visible changes with temperature and that undergo phase transitions. While compelling and promising, this method in new, and should be further developed by relying on established source for information in parallel is advised.

## Data Availability

The datasets generated and/or analysed during the current study are available in the Mendeley Data repository, [https://data.mendeley.com/datasets/wsvn7pdc5y/3]
